# Non-redundant functions of FAK and Pyk2 in intestinal epithelial repair

**DOI:** 10.1038/s41598-019-41116-1

**Published:** 2019-03-14

**Authors:** Keena S. Thomas, Katherine A. Owen, Kathryn Conger, Ryan A. Llewellyn, Amy H. Bouton, James E. Casanova

**Affiliations:** 1 University of Virginia School of Medicine, Department of Microbiology, Immunology and Cancer, Charlottesville, VA 22908 USA; 2 University of Virginia School of Medicine, Department of Cell Biology, Charlottesville, VA 22908 USA; 3 Present Address: Ampel Biosolutions, Charlottesville, VA 22902 USA; 4 Present Address: La Jolla Institute for Allergy and Immunology, La Jolla, CA 92037 USA

## Abstract

Adhesion signaling between epithelial cells and the extracellular matrix plays a critical role in maintaining tissue homeostasis and the response to tissue damage. Focal adhesion kinase (FAK) and its close relative Pyk2 are non-receptor tyrosine kinases that mediate adhesion signaling to promote cell proliferation, motility and survival. FAK has also been shown to act as a mechanosensor by modulating cell proliferation in response to changes in tissue compliance. We previously showed that mice lacking FAK in the intestinal epithelium are phenotypically normal under homeostatic conditions but hypersensitive to experimental colitis induced by dextran sulfate sodium (DSS). Here we report that Pyk2-deficient mice are also phenotypically normal under homeostatic conditions and are similarly hypersensitive to DSS-induced colitis. These data indicate that normal intestinal development and homeostatic maintenance can occur in the presence of either FAK or Pyk2, but that both kinases are necessary for epithelial repair following injury. In contrast, mice lacking both FAK and Pyk2 develop spontaneous colitis with 100% penetrance by 4 weeks of age. Normal colonic phenotype and function are restored upon treatment of the double knockout mice with antibiotics, implicating commensal bacteria or bacterial products in the etiology of the spontaneous colitis exhibited by these mice.

## Introduction

The mammalian intestinal tract serves as a dynamic physical barrier segregating the luminal microbial community from underlying mucosal tissue. Under homeostatic conditions, the intestinal epithelium undergoes rapid self-renewal, turning over every 4–5 days. During this process, intestinal stem cells (ISCs) residing near the crypt base give rise to rapidly proliferating transit amplifying cells, which then differentiate into enterocytes, goblet cells, enteroendocrine cells, tuft cells and Paneth cells^1,2^. The maintenance of intestinal homeostasis also depends on the symbiotic relationship existing between the gut microbiota and host. It is now well established that the intestinal microflora play a key role in modulating nutrient metabolism and absorption, controlling intestinal development and function^3^, and shaping the gastrointestinal immune landscape^4^.

Mucosal erosion or injury results in the rapid expansion of proliferative cells and their subsequent maturation to restore normal tissue architecture and function^5^. During mucosal injury, commensal microbes and microbial products penetrate the epithelial barrier, resulting in the recruitment and activation of innate and adaptive immune cells^6^. Activated macrophages, dendritic cells, and T lymphocytes located within the lamina propria produce numerous inflammatory cytokines that both promote intestinal inflammation and contribute to the regenerative response. Impaired barrier function is a hallmark of chronic inflammatory bowel disorders (IBD) including Crohn’s disease (CD) and ulcerative colitis (UC)^7,8^.

Focal Adhesion Kinase (FAK) and the related protein Proline-rich Tyrosine Kinase 2 (Pyk2) are non-receptor tyrosine kinases best known for their roles in adhesion signaling, survival and migration where they transmit adhesion-dependent signals to the cell interior^9,10^. Despite their overall structural similarity, there are several key differences between the two kinases. In many cell types, FAK has an important role in mechanotransduction, effectively acting as a sensor of tissue compliance^11–15^. Previous work from our group^16^ and others^17^ has shown that the ability of FAK to sense mechanical changes that occur in response to inflammation-induced collagen deposition is critical to intestinal epithelial repair. However, a similar role for Pyk2 in mechanotransduction has not been established. While both FAK and Pyk2 can be activated following integrin mediated adhesion, Pyk2 is primarily activated in response to a variety of stimuli that increase intracellular calcium^18–22^. In addition, Pyk2 exhibits a limited tissue distribution compared to FAK, with expression being highest in cells of hematopoietic lineage and in the central nervous system^19,23,24^. Interestingly, the intestinal epithelium is one of only a few tissues that co-express both FAK and Pyk2^16^, prompting the question of whether these molecules share common functions in regulating normal homeostasis and repair in the intestine, or whether they have distinct functions that act in tandem to control tissue integrity.

In this study, we examine for the first time the role of Pyk2 in intestinal homeostasis and repair using Pyk2-deficient mice (hereafter Pyk2^−/−^). Similar to our earlier findings in FAK-deficient animals^16^, Pyk2^−/−^ animals did not exhibit any intestinal dysfunction in the absence of injury, indicating that intestinal morphogenesis and homeostasis can occur normally when either one of these kinases is present. However, as was the case for FAK, mice lacking Pyk2 exhibited a marked hypersensitivity to chemically-induced colitis, characterized by pronounced edema, extensive mucosal ulceration, and crypt loss. Interestingly, epithelial-specific loss of FAK (FAK^∆IEC^) combined with global loss of Pyk2 (FAK^∆IEC^/Pyk2^−/−^) resulted in a pronounced phenotype in the absence of any perturbation culminating in a spontaneous fatal colitis at 4 weeks of age with 100% penetrance. Antibiotic treatment at the time of weaning was sufficient to rescue this phenotype, implicating microbiota-induced inflammation in the onset and/or resolution of this severe pathology. Together, these findings suggest that FAK and Pyk2 contribute unique and/or additive functions that are essential for epithelial repair under conditions of significant perturbation. However, during homeostasis when the need for epithelial repair is significantly reduced, either molecule alone has the capacity to provide the necessary functions required for maintenance of colonic integrity.

## Results

### FAK and Pyk2 have unique and/or additive roles in intestinal function

In an earlier study, we generated intestinal epithelial cell (IEC)-specific FAK knockout mice (FAK^ΔIEC^) using a villin-Cre driver^16^. In the absence of injury, these animals were phenotypically identical to wildtype (WT) controls, with normal villus architecture, epithelial polarity, and expression/localization of tight junction and adherens junction proteins^16^. However, these mice were profoundly susceptible to experimental colitis induced by oral administration of dextran sulfate sodium (DSS). Injury in FAK^ΔIEC^ mice was characterized by elevated p53 expression, increased sensitivity to apoptosis, and most notably, an almost complete failure to upregulate epithelial cell proliferation during the repair response^16^.

**Figure 1:**
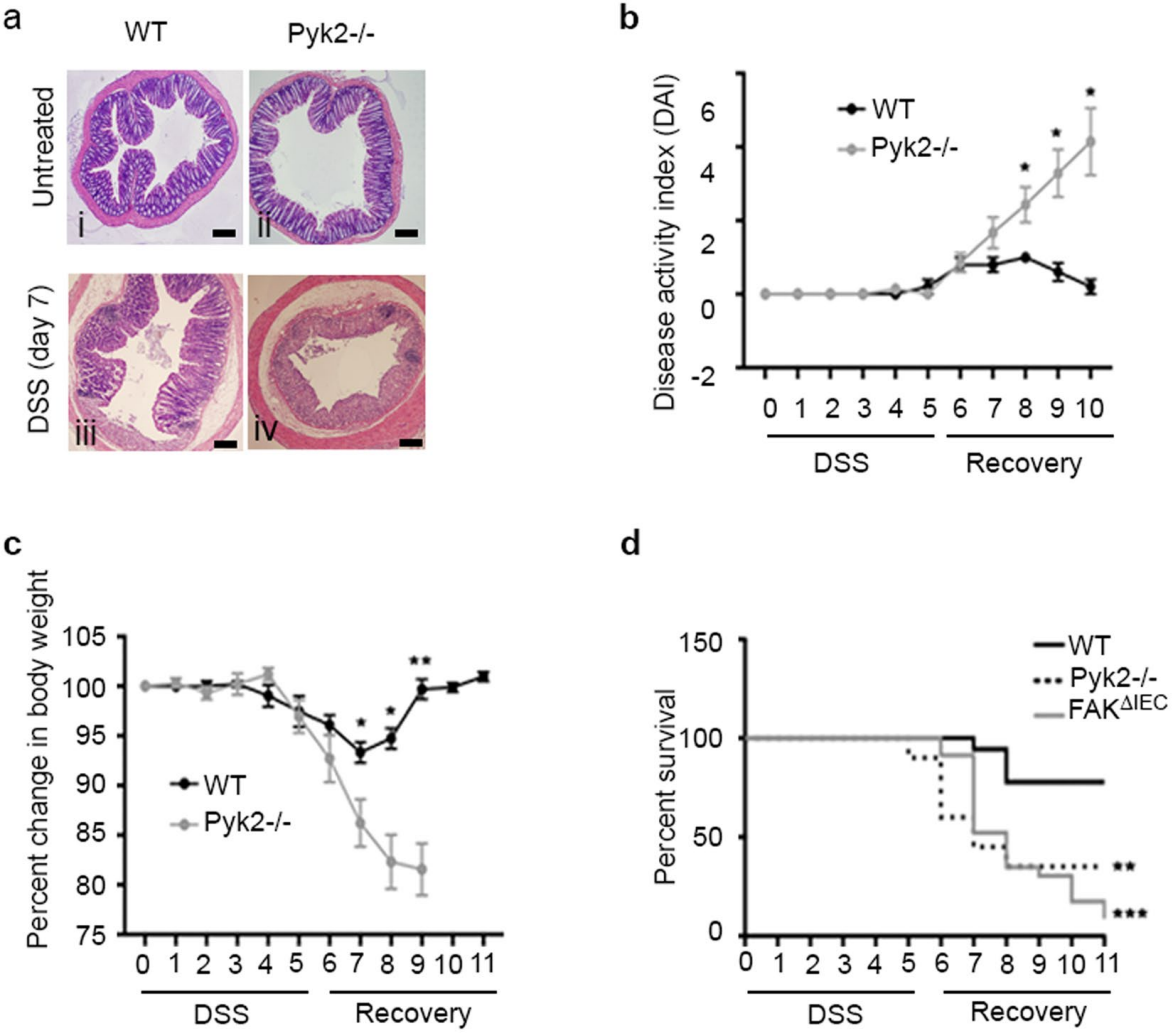
Pyk2−/− mice are hypersensitive to DSS treatment. (**a**) Low magnification (4x) representative H&E stained colon sections generated from untreated and DSS treated WT and Pyk2^−/−^ mice. Scale bars represent 200 μm. The mean change in disease activity index (**b**) and body weight (**c**) are shown for WT (N = 5) and Pyk2^−/−^ (N = 7) DSS-treated mice. Asterisks indicate values that are significantly different from WT mice at the same time point (*P < 0.05, **P < 0.001). In (**b**) D8 P = 0.0249, D9 P = 0.0075, D10 P = 0.0045. In (**c**) D7 P = 0.0259, D8 P = 0.0030, D9 P = 0.0002. Two-tailed t-test (**d**) Percent survival of WT, Pyk2^−/−^ and FAK^ΔIEC^ mice as a function of time (days).

As mentioned above, the only other member of the FAK family of nonreceptor protein tyrosine kinases, Pyk2, is also expressed in IECs^16^. Mice constitutively lacking Pyk2 (Pyk2^−/−^) develop normally^25^ and exhibit normal colonic architecture and proliferation (Fig. 1a, panels i and ii, and Supplementary Fig. S1, panel c), although the crypt length and number of cells per crypt are slightly reduced in the Pyk2^−/−^ animals (Supplementary Fig. S1, panels a and b). However, given the hypersensitivity of FAK^ΔIEC^ mice to DSS-induced injury, we questioned whether the loss of Pyk2 would result in a similar hypersensitivity to experimental colitis. To test this, WT and Pyk2^−/−^ animals were given 2.5% DSS in their drinking water for 5 days, followed by a 5–6 day recovery period during which the clinical course of disease was observed. By day 7 (5 days DSS, 2 days recovery), the colonic epithelium of WT controls remained largely intact, but exhibited patchy ulceration, edema, and evidence of epithelial regeneration adjacent to ulcerated areas (Fig. 1a, panel iii). More severe tissue damage was apparent in the Pyk2^−/−^ mice at day 7, characterized by pronounced edema, extensive mucosal ulceration, and almost complete loss of normal crypt architecture (Fig. 1a, panel iv). In agreement with histological observations, DSS-treated Pyk2^−/−^ mice exhibited significantly higher disease activity scores, driven largely by gross rectal bleeding and diarrhea (Fig. 1b). All of the treated animals lost weight during the treatment protocol; however, while WT mice recovered their weight by day 9, the Pyk2^−/−^ animals continued to experience severe weight loss and were typically euthanized on day 9 due to a 20% reduction in weight (Fig. 1c). This correlated with increased mortality for Pyk2^−/−^ animals, similar to that observed in FAK^ΔIEC^ mice. (Fig. 1d). Together, these data suggest that, while FAK and Pyk2 appear to play redundant roles in the maintenance of colonic homeostasis, they have unique functions with respect to mucosal recovery after damage. Although we consider it unlikely, we cannot exclude the alternative explanation that a threshold level of FAK and/or Pyk2 expression/activity is required for epithelial repair that is not reached when one or the other molecule is genetically deleted from colonic epithelial cells.

### Mice lacking both FAK and Pyk2 develop spontaneous colitis

**Figure 2:**
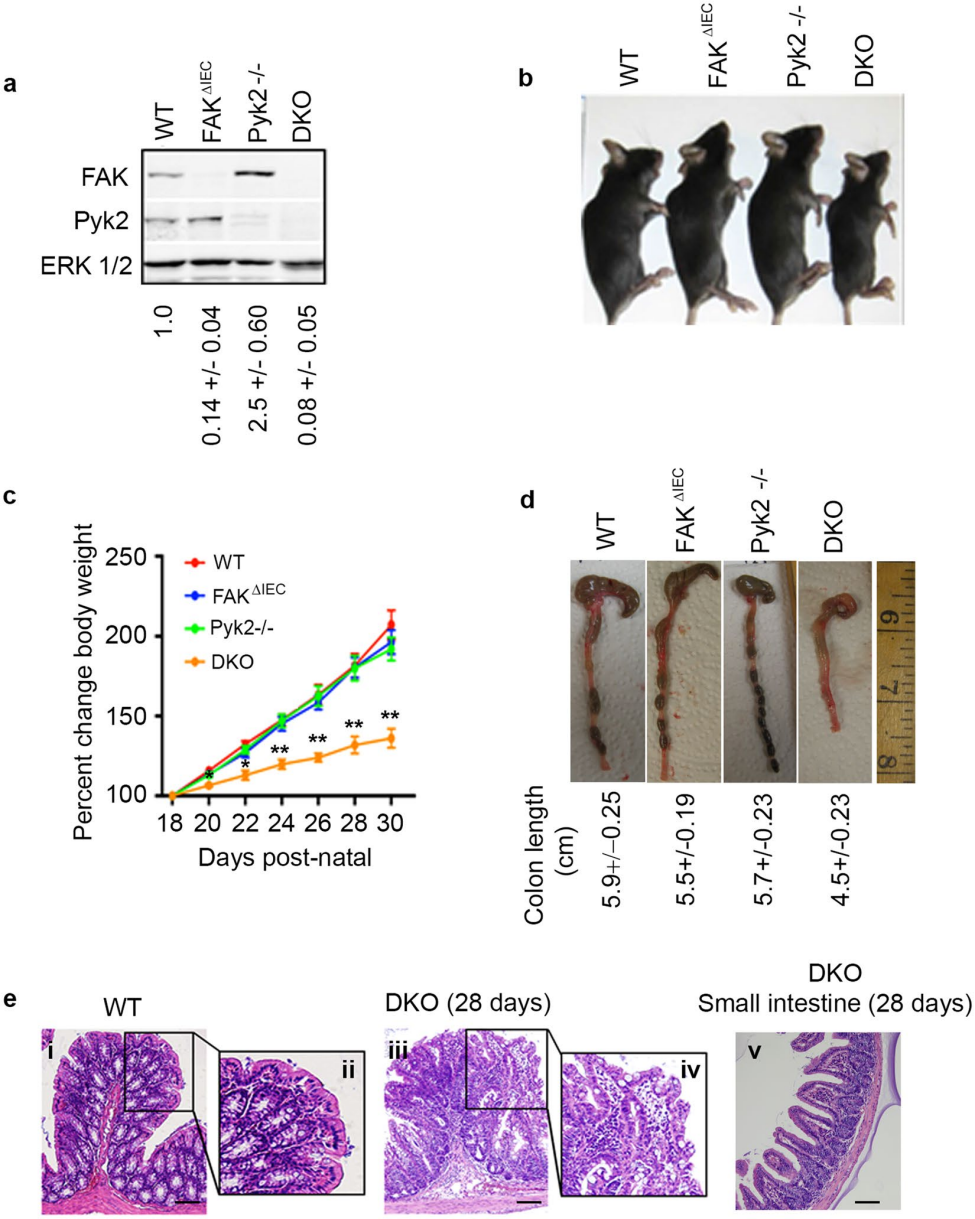
DKO mice exhibit a severe spontaneous colitis in the absence of external challenge. (**a**) Immunoblot analysis of primary colon epithelial cells isolated from WT, FAK^ΔIEC^, Pyk2^−/−^ and DKO mice. Densitometry of immunoblots from 3 independent experiments was analyzed using ImageStudio (ver. 5.2) software. The values shown below each lane represent the ratio of FAK to ERK (loading control) relative to the ratio obtained for WT samples. (**b**) Representative images of 30-day old WT, FAK^ΔIEC^, Pyk2^−/−^ and DKO mice. (**c**) The mean percent change in body weight is shown for WT (N = 6), FAK^ΔIEC^ (N = 8), Pyk2^−/−^ (N = 5) and DKO (N = 8) mice. Asterisks indicate DKO values that are significantly different from WT on the same day (*P < 0.05, **P < 0.001). D20 P = 0.0406, D22 P = 0.0004, D24 P = 9.7E-06, D26 P = 7.4E-06, D28 P = 6.3E-06, D30 P = 2.3E-05. Two-tailed t-test (**d**) Representative images of colons isolated from 30 day old mice. Average colon lengths were obtained from WT (N = 6), FAK^ΔIEC^ (N = 7), Pyk2^−/−^ (N = 5) and DKO (N = 8) mice. (**e**) Representative 10X H&E staining of WT (i) and DKO (iii) colon sections, showing crypt architecture. Scale bars represent 100 μm. Panel v shows a representative image of the small intestine of a DKO mouse at 28 days.

To further understand the roles played by FAK and Pyk2 in intestinal function, we crossed FAK^ΔIEC^ and Pyk2^−/−^ mice to generate double knock out animals (DKOs). Protein analysis from primary colon epithelial cell lysates confirmed the specificity of FAK and Pyk2 deletion (Figs 2a and S2). Quantification of FAK revealed an elevated level of expression in colonic epithelial cells isolated from the Pyk2^−/−^ mice. Interestingly, in contrast to FAK^ΔIEC^ and Pyk2^−/−^ single knockout mice, DKO animals exhibited a marked phenotype in the absence of external challenge. These mice were significantly smaller than WT littermate controls by 3 weeks of age (WT = 7.4 +/− 0.94 g, FAK^∆IEC^ = 7.7 +/− 0.63 g, Pyk2^−/−^ = 8.0 +/− 0.4 g, DKO = 6.6 +/− 0.99 g) (Fig. 2b), and differences in body weight became more pronounced over the ensuing 10 days, suggesting a defect in intestinal function (Fig. 2c). Gross examination of intestinal tissue revealed that the colons of the DKO mice were free of solid fecal pellets and shorter than those of WT, FAK^ΔIEC^, or Pyk2^−/−^ mice, both well-established hallmarks of colitis (Fig. 2d). Histological examination of DKO colons revealed a strikingly disorganized crypt architecture with evidence of aberrant crypt branching, loss of goblet cells, and the presence of an inflammatory infiltrate, consistent with the inability of these mice to gain weight (Fig. 2e, panels i-iv). In contrast to the colon, the architecture of the small intestine in DKO mice appeared completely normal (Fig. 2e, panel v, and Supplementary Fig. S3.) This spontaneous colitis was evident in 100% of DKO mice, and all animals were euthanized by post-natal day 28–32 due to the severity of disease.

**Figure 3:**
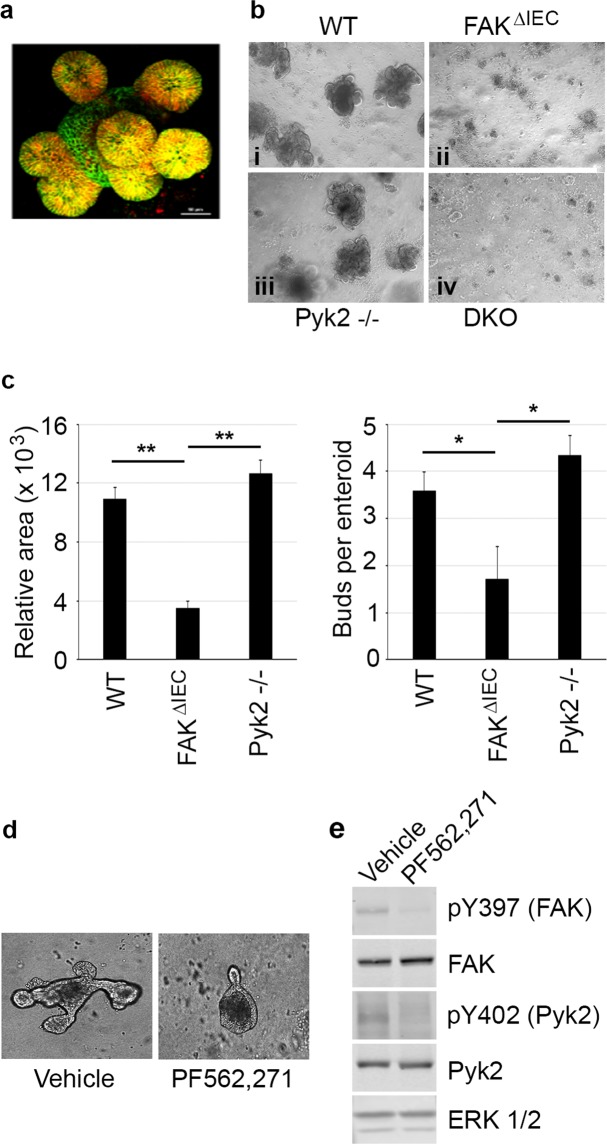
Budding of intestinal organoids is dependent on FAK but not Pyk2. (**a**) Confocal stack of a single WT organoid cultured for 10 days showing E-cadherin expression (green) throughout the organoid, and CD44 (marker for proliferating crypt cells; red) in cells present in the buds (small spherical structures appearing yellow in the image). Bar represents 50 μm. (**b**) Intestinal crypts were harvested from WT (i), FAK^ΔIEC^ (ii), Pyk2^−/−^ (iii) or DKO (iv) mice treated with antibiotics in their drinking water, and grown for 9 days in culture. (**c**) Quantification of enteroid cultures. Left panel: Relative area was calculated using ImageJ (NIH) for WT (N = 19), FAK^ΔIEC^ (N = 7) and Pyk2^−/−^ (N = 18) enteroids. Asterisks indicate values that are significantly different based on a two-tailed t-test for WT compared to Fak^ΔIEC^ (*P* = 3.1E-08) and Fak^ΔIEC^ compared to Pyk2^−/−^ (*P* = 1.3E-08). Right panel: Buds per enteroid were counted for WT (N = 20), FAK^ΔIEC^ (N = 7) and Pyk2^−/−^ (N = 18). Asterisks indicate values that are significantly different based on a two-tailed t-test for WT enteroids compared to FAK^ΔIEC^ enteroids (*P* = 0.014) and FAK^ΔIEC^ compared to Pyk2^−/−^ (*P* = 0.0016). (**d**) Intestinal crypts were harvested from WT mice and treated with vehicle (DMSO) or 1 μM of the dual FAK/Pyk2 inhibitor PF 562,271 one day after suspension in Matrigel. Cultures were maintained in continuous presence of vehicle or drug for 7 days. (**e**) Representative immunoblot containing proteins isolated from WT organoids treated with vehicle or PF 562,271 for 1 hour.

In light of the severe spontaneous colitis exhibited by DKO mice, we next used an enteroid model of *ex vivo* epithelial regeneration^26^ to determine whether there was a cell-intrinsic defect in the epithelial cells that prohibited normal colonic morphogenesis. This was particularly important due to the global nature of the Pyk2 knockout, since a contribution of Pyk2 loss in stromal cells could not be exluded. Because murine colonic enteroids are extremely challenging to grow, we elected to generate enteroids from small intestines of mice from each genotype. Irrespective of the source of crypt cells, this approach provides a means to examine epithelial cell-intrinsic proliferative capacity. Small intestinal crypts were isolated from WT, FAK^∆IEC^, Pyk2^−/−^, and DKO mice, embedded in matrigel, and cultured in standard enteroid media containing epidermal growth factor (EGF), Noggin, and R-spondin1 (EGR). Over several days, WT crypts sealed to form a hollow lumen and developed numerous new crypt-like buds containing proliferating stem cells (Fig. 3a,b, panel i). Enteroids derived from Pyk2-deficient crypts developed as efficiently as WT crypts, while enteroids from FAK^∆IEC^ mice were dramatically reduced in numbers and did not develop new buds (Fig. 3b, panels ii and iii, Fig. 3c). Crypts isolated from DKO mice failed to generate enteroids at all, and were therefore not quantified (Fig. 3b, panel iv). These data suggest that FAK and Pyk2 have distinct cell-intrinsic functions under these culture conditions, wherein FAK is essential for enteroid growth while Pyk2 is not.

To determine whether the inability of FAK/Pyk2 knockout cells to form organoids was dependent upon the kinase activity of these molecules, crypts were isolated from WT mice and allowed to form spheroids before treatment with 1 μM PF 562,271, a pharmacological inhibitor of both FAK and Pyk2 kinase activity^27,28^. The prominent budding that was evident in vehicle-treated control spheroids by day 7 was markedly absent in the drug-treated spheroids (Fig. 3d). Immunoblots on cell extracts isolated from the cultures confirmed that PF 562,271 abrogated the activity of both FAK and Pyk2 (Figs 3e and S4). Together, these data indicate that FAK/Pyk2 activity is required to promote the development of enteroids under these conditions.

### Microbiota-induced inflammation contributes to spontaneous colitis in DKO mice

**Figure 4:**
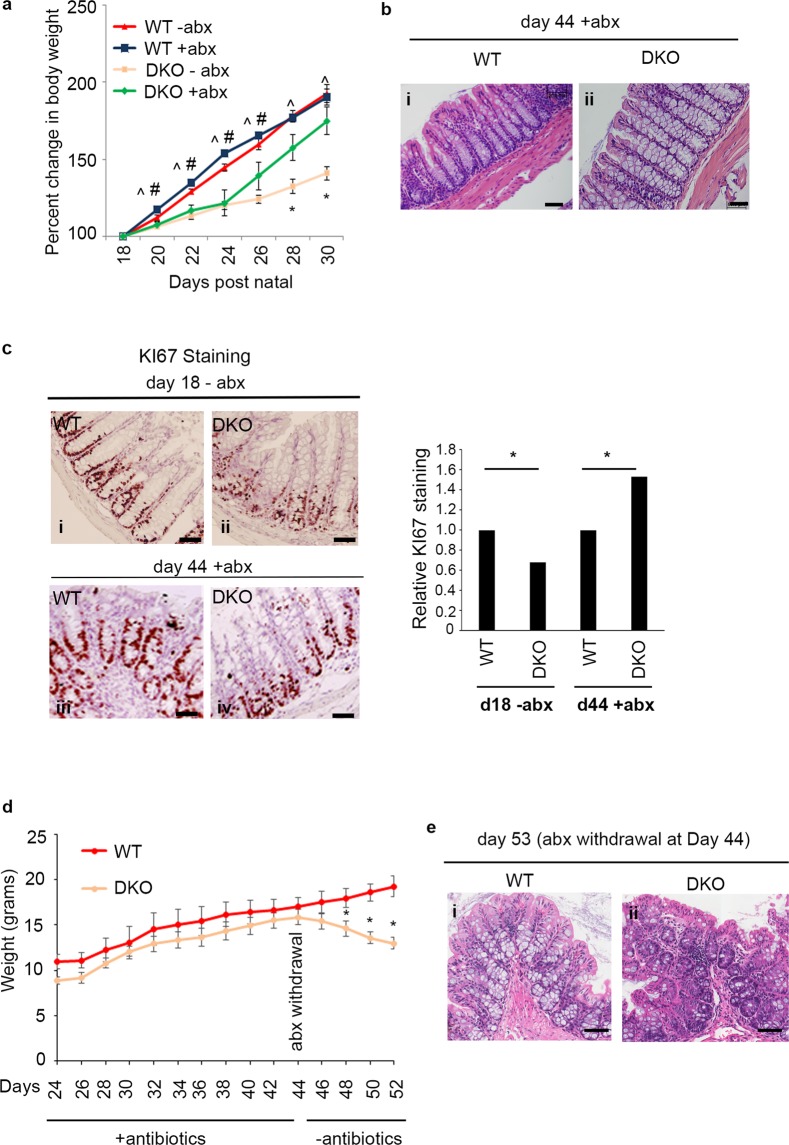
Antibiotic treatment of DKO mice results in weight gain and restoration of normal colon morphology. (**a**) Mean percent change in body weight is shown for WT mice in the absence of antibiotic treatment (WT -abx) (N = 6), WT + abx (N = 3), DKO – abx (N = 8), and DKO + abx (N = 5) mice. ^Indicates DKO-abx values that are statistically different from WT-abx. ^#^Indicates DKO + abx values that are statistically different from WT + abx. *Indicates DKO-abx values that are statistically different from DKO + abx. In all cases, P < 0.05. (**b**) Representative H&E staining of WT (i) and DKO (ii) colons of mice treated with antibiotics (ciprofloxacin/metronidazole). Antibiotics were administered when the mice were weaned at day 23, and tissues were harvested at day 44. Bars represent 50 μm. (**c**) Colon sections were obtained from untreated WT (i) and DKO (ii) mice at day 18, and from WT (iii) and DKO (iv) mice treated with antibiotics from day 23 to day 44. Tissues were stained for KI67 and the percentage of stained cells per intact crypt was enumerated for each condition. Bars represent 50 μm. Data shown are the relative numbers of positively stained cells obtained from approximately 35–45 crypts. Statistics were performed using the 2-sample z-test to compare sample proportion. Asterisks indicate P < 0.0001 (**d**) WT (N = 5) and DKO mice (N = 5) were given ciprofloxacin/metronidazole in their drinking water from day 23 through day 44, after which the antibiotics were withdrawn. Data shown are the mean body weight at each time point. Asterisks indicate values that are significantly different at that day (*P < 0.05). ^D20 P = 0.0406, ^Day 22 P = 0.0003, ^Day 24 P = 9.7E-06, ^Day 26 P = 7.4E-06, ^Day 28 P = 6.3E-06, ^Day 30 P = 2.3E-05, ^#^Day 20 P = 0.0070, ^#^Day 22 P = 0.0048, ^#^Day 24 P = 0.0170, ^#^Day 26 P = 0.0416, *Day 28 P = 0.0363, *Day 30 P = 0.0143. Two-tailed t-test. (**e**) Representative colon sections from WT (i) and DKO (ii) mice sampled at day 53 after removal from antibiotic treatment at day 44. Scale bars represent 50 μm.

As mentioned above, histological examination of the small intestine of DKO mice revealed normal crypt/villus architecture with no signs of inflammation despite the fact that the colons revealed marked pathology (Fig. 2e, panel v). We hypothesized that the presence of higher levels of commensal bacteria in the colon coincident with leakage of either intact bacteria or their products into the underlying mucosa could potentially account for the spontaneous colitis observed in these mice. In support of this hypothesis, treatment of DKO mice with oral antibiotics (ciprofloxacin/metronidazole) beginning  when the mice were weaned at day 23 resulted in an almost immediate increase in weight gain (Fig. 4a) and a corresponding restoration of normal colon morphology and cell proliferation (Fig. 4b,c). To determine whether antibiotic treatment conferred a permanent restitution of intestinal integrity under homeostatic conditions, antibiotics were withdrawn at day 44. This resulted in an immediate weight loss in the DKO mice and the reemergence of epithelial disruption and inflammation (Fig. 4d,e). These data suggest that the combined loss of FAK and Pyk2 increases sensitivity to the presence of the commensal microbiota, leading to severe colonic inflammation and dysfunction.

## Discussion

Both FAK and Pyk2 are important transmitters of adhesion-dependent signals that promote motility, proliferation and survival in metazoans^9,18,29,30^. It is well established that FAK can act as a mechanosensor, enhancing cell proliferation in response to changes in tissue compliance^11–13,31^. Pyk2 has also been shown to respond to changes in mechanical stress, but whether it does so directly or indirectly is less clear^32,33^. We recently demonstrated that FAK and Pyk2 are co-expressed in the intestinal epithelium, raising the question of whether the two proteins have redundant or distinct functions in this tissue^16^. In that earlier study, we showed that mice lacking FAK (but expressing endogenous Pyk2) in the intestinal epithelium undergo morphogenesis normally and exhibit normal intestinal morphology under homeostatic conditions. However, these mice displayed hypersensitivity to DSS-induced colitis that resulted from a dramatic attenuation of cell proliferation in response to injury^16^. This result suggested that Pyk2 is not sufficient to promote colonic epithelial repair.

In the current study, we tested this hypothesis directly using Pyk2-deficient mice. We found that Pyk2^−/−^ mice were phenotypically similar to FAK-deficient mice, exhibiting normal intestinal morphogenesis and homeostatic maintenance, but also exhibiting extreme hypersensitivity to DSS-induced colitis. These observations suggest that FAK and Pyk2 may have overlapping roles in intestinal morphogenesis and maintenance but have non-redundant functions in the context of epithelial regeneration.

To determine if these non-redundant functions are intrinsic to epithelial cells, we used *ex vivo* enteroid cultures comprised solely of intestinal epithelial cells, without any contribution from underlying stromal cells. Somewhat surprisingly, we found that enteroids derived from Pyk2-deficient mice were phenotypically indistinguishable from WT, with numerous proliferative crypt-like buds. In contrast, enteroids derived from FAK-deficient or DKO mice exhibited dramatically attenuated growth. Moreover, sealed crypts treated with the dual FAK/Pyk2 inhibitor PF562,271 failed to undergo the budding/proliferative program typically seen in WT cultures. Together, these data indicate that FAK plays a critical role in the proliferative response of enteroids under these culture conditions.

The growth of enteroids in culture requires a cocktail of growth factors, including EGF, R-spondin and Noggin^34–36^. It is therefore possible that FAK, but not Pyk2, mediates signaling through one or more of these molecules in intestinal epithelial cells. Both FAK and Pyk2 have been linked to signaling via EGF receptors^37,38^, but whether they act upstream (to transactivate) or downstream as effectors appears to vary with cell type and stimulus. We are currently investigating whether impaired EGFR signaling underlies the failure of FAK-deficient cells to proliferate in this setting. This possibility is particularly compelling in light of the established role of FAK as a mechanotransducer, exhibiting low activity on soft substrates and increased activity on stiffer ones^12^. We have previously shown that cultured colonic epithelial cells proliferate more rapidly on stiff matrices (4800 pascals) than on softer ones (300 pascals) but that this difference is abrogated when cells are depleted of FAK^16^. In agreement with these observations, a recent study reported that the mechanosensing properties of FAK are linked to intestinal epithelial regeneration through activation of the Hippo signaling pathway^17^. This study demonstrated that the Hippo pathway and its transcriptional effectors YAP/TAZ are activated by remodeling of the extracellular matrix in response to colonic injury. Specifically, collagen deposition, which is a hallmark of colitis, stimulates activation of FAK, leading to downstream activation of YAP/TAZ. Through transcriptional reprogramming, YAP/TAZ then promote the expansion of a highly proliferative pool of fetal-like epithelial cells that repopulate the damaged epithelium^17^.

While loss of either FAK or Pyk2 has no significant effect on intestinal morphogenesis or homeostatic maintenance, simultaneous loss of both kinases leads to a fully penetrant, spontaneous (and ultimately fatal) colitis in the absence of induced injury. Somewhat surprisingly, treatment of mice with antibiotics restored normal colonic morphology, with a corresponding increase in weight gain and prolonged survival. This finding underscores the requirement for intestinal microbiota in the generation of the severe colitis exhibited by the DKO mice. It also shows that, in the absence of microbiota, the development of the colonic epithelium and its maintenance does not require either FAK or Pyk2. One possible explanation for this finding is that epithelial barrier integrity becomes compromised when both FAK and Pyk2 are absent, causing leakage of microbial products across the epithelium that induce the robust inflammation we observe in the absence of antibiotics. The ensuing changes in the physical properties of the damaged epithelium (e.g. collagen deposition) that would otherwise induce activation of a robust proliferative burst would be severely compromised in the absence of FAK.

Conversely, barrier integrity may not be directly compromised by dual loss of FAK and Pyk2. Instead, the DKO mice may be abnormally hypersensitive to the small amounts of leakage across the epithelium that are inherent to the normal homeostatic process. In this case, loss of Pyk2 may contribute to an augmented inflammatory response that results in increased fibrosis and tissue damage, particularly in light of the global nature of the Pyk2 knockout mice used for these studies and the impaired function reported for Pyk2^−/−^ macrophages^25,39^ Loss of FAK in the colonocytes would then augment the severity of the phenotype by impairing the repair process as described above. This is consistent with our finding that intestinal epithelial cells are capable of establishing an appropriate proliferative program in enteroid cultures *ex vivo* in the absence of Pyk2 but not in the absence of FAK. This model could be definitively tested in the future with the establishment of conditional macrophage- and intestinal epithelial-specific Pyk2 knockout mice. Finally, it is formally possible that FAK and Pyk2 share some redundancy in function and, in the absence of a threshold activity of these activities, a small microbial challenge could result in a highly magnified response.

## Methods

### Mice

Constitutive Pyk2 knockout (KO) mice **(**Pyk2^−/−^) in a C57Bl/6 background were kindly provided by Dr. Brian Bond and Pfizer, Inc^39^. Intestinal epithelial cell (IEC)-specific FAK knockout mice (FAK^ΔIEC^) previously generated by our group^16^, also in a C57/Bl/6 background, were crossed with Pyk2^−/−^ mice to generate villin^WT/Cre^FAK^f/f^Pyk2^−/−^ mice, hereafter designated double knockout (DKO).

### DSS treatment

12 week old mice were given 2.5% dextran sulfate sodium (DSS; MP Biomedicals, LLC. Solon, OH) in their drinking water for 5 days, after which the mice were provided normal water. Disease activity index was calculated as previously described^16^. Following euthanasia, colons were excised, measured and processed for analysis. All animal experiments were approved by the University of Virginia Institutional Animal Care and Use Committee (IACUC), and were conducted in accordance with the relevant guidelines and regulations.

### Antibiotic treatment

At the time of weaning, mice treated with antibiotics were provided drinking water containing 0.66 mg/ml ciprofloxacin (Sigma-Aldrich) and 2.5 mg/ml metronidazole (Sigma-Aldrich) and 60 g/L sucrose. Control mice were provided water supplemented with sucrose but no antibiotics. Mice were weighed daily and observed for evidence of discomfort and disease. Disease activity index (DAI) was calculated as described previously^16^. Following antibiotic treatment, colons were excised, measured and processed for analysis.

### Histology and immunostaining

Intestinal tissues were processed, formalin-fixed and paraffin-embedded as described previously^16^. Hematoxylin and eosin (H&E) staining was performed by the UVA Histology Core. KI67 staining was performed as previously described^16^ using Anti-Mouse KI-67 from DakoCytomation (Denmark). Sections were examined with an Olympus BX51 microscope and images were acquired with an Olympus DP70 digital camera controlled by ImagePro Plus software (EPIX, inc. Buffalo grove, IL), as previously described^40,41^.

### Morphometry

Measurements of villus and crypt length were obtained using Image J (NIH) software. Comparisons were made between paired samples using the two-tailed t-test. Cells per crypt were enumerated by counting nuclei.

### Antibodies and reagents

The antibodies used in these studies were obtained from the following vendors: polyclonal anti-pFAK^pY397^, polyclonal anti-pPyk2^pY402^, and monoclonal anti-Pyk2 (BD Transduction Laboratories, San Jose, CA); polyclonal FAK C-20 (Santa Cruz Biotechnology, Inc., Santa Cruz, CA); polyclonal anti-ERK1/2 (Cell Signaling, Danvers, MA). EGF, Noggin and R-spondin were obtained from R and D Systems (Minneapolis, MN).

### Enteroid culture

Intestines were dissected from 6–8 week old mice, flushed with ice cold PBS and cut into 3 mm pieces. Fragments of small intestine were incubated in ice cold chelation buffer (2 mM EDTA) for 30 minutes. After removal of the chelation buffer, the tissues were placed in Shaking Buffer (PBS, 43.3 mM Sucrose, 54.9 mM Sorbitol) and rocked for 2 minutes. Crypts were filtered through a 70 μm filter to remove larger fragments, then resuspended in 50 μL of Matrigel (BD Bioscience. San Jose, CA) containing 50 ng/mL EGF, 100 ng/mL Noggin and 500 ng/mL R-spondin. Matrigel was allowed to polymerize fully in a 37 °C incubator for 30–90 minutes, then overlaid with DMEM/F12 culture media (Invitrogen) containing L-glutamine, penicillin/streptavidin, 10 mM Hepes, N2 supplement (1:100), and B-27 supplement (1:50) all from Invitrogen. For whole mount staining of enteroids and confocal imaging, the enteroids were fixed with 2% paraformaldehyde at room temperature for 30 minutes. Fixation was quenched with 50 mM NH_4_Cl for 30 minutes. The cultures were permeabilized with 0.5% Triton X-100 for 30 minutes and then blocked with 3% BSA in PBS for 1 hour at room temperature. Samples were incubated with primary antibodies against E-cadherin and CD44 (both from Cell Signaling) overnight at 4 °C. The next day, cultures were washed with PBST (PBS + 0.05% Tween) twice for 5 minutes, then incubated with secondary antibodies overnight at 4 °C. Nuclei were stained with 2 μg/mL Hoescht for 20 minutes. Images were acquired on a Nikon C1 Plus confocal microscope and 3-dimensional image stacks (0.5 µm sections) were flattened into a single image using Nikon Elements software. When enteroids were treated with inhibitors, drugs were added one day after suspension in Matrigel, and maintained in the culture for the duration of the experiment.

## Supplementary information

Supplementary data

## Data Availability

All data generated or analyzed during this study are included in the published article and its supplementary information files. Any and all reagents, including mice, that are not commercially available will become publicly available upon publication.
