# Supplementary material for: Non-redundant functions of FAK and Pyk2 in intestinal epithelial repair
Source: Sci Rep. 2019 Mar 14;9:4497. doi: 10.1038/s41598-019-41116-1 (PMC6418130; doi:10.1038/s41598-019-41116-1)
Supplement: Supplementary file 1 — Supplementary data [file 41598_2019_41116_MOESM1_ESM.pdf]

Supplementary Information:

Non-redundant functions of FAK and Pyk2 in intestinal epithelial repair

Keena S. Thomas[1], Katherine A. Owen[2,3], Katherine Conger[2], Ryan A. Llewellyn[1,4], Amy H. Bouton[1*] and James E. Casanova[2*]

[1]University of Virginia School of Medicine, Department of Microbiology, Immunology and Cancer, Charlottesville, VA 22908, USA

[2] University of Virginia School of Medicine, Department of Cell Biology, Charlottesville, VA 22908, USA

[3] Current address, Ampel Biosolutions, Charlottesville, VA 22908, USA

[4]Current address, La Jolla Institute for Allergy and Immunology, La Jolla, CA 92037, USA

*Corresponding authors: ahb8y@virginia.edu, jec9e@virginia.edu

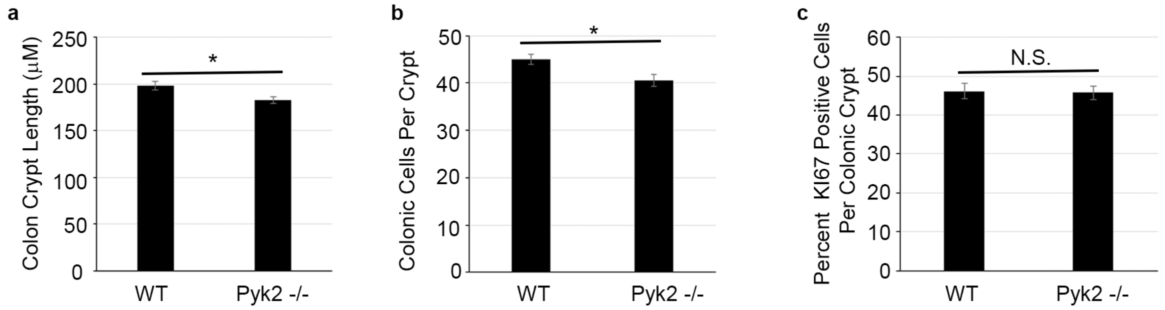

Supplementary Fig. S1. WT and Pyk2$^{-/-}$ mice exhibit small differences in crypt length and cell content. (a) Measurement of colon crypt length in WT (61 crypts) and Pyk2$^{-/-}$ (49 crypts) mice using Image J (NIH). *P* = 0.0128 (two tailed t-test). (b) Enumeration of cells per crypt, counting 40 crypts per genotype. *P* = 0.0103 (two tailed t-test). (c) Colon sections were obtained from WT and Pyk2$^{-/-}$ mice. Tissues were stained for KI67 and the percentage of stained cells per intact crypt was enumerated for each genotype. Data shown are from 40 crypts per genotype. *P*=0.8876 (two tailed t-test).

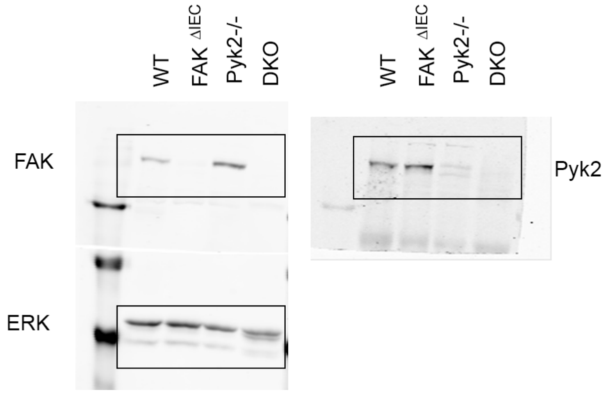

Supplementary Fig. S2. Original immunoblots corresponding to Figure 2. Primary colonocytes were harvested from the indicated mice, lysed immediately, and immunoblotted for either FAK (left) or Pyk2 (right). Equivalent loading was confirmed by probing for ERK. Areas that were cropped and used in Fig. 2 are boxed.

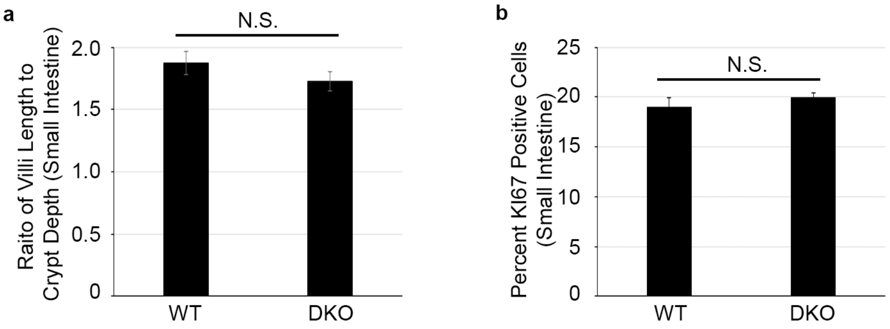

Supplementary Fig. S3. WT and DKO mice exhibit no statistically significant differences in small intestinal architecture or crypt cell proliferation. (a) Measurements of villus length and crypt depth were obtained using Image J (NIH). The ratio of villus length to crypt depth was calculated for 20 crypts from WT and DKO mice. *P*=0.3873 (two tailed t-test). (b) Small intestines were harvested from 28 day-old WT and DKO mice. Tissues were stained for KI67 and the percentage of stained cells per crypt was enumerated for each genotype. Data shown are from 20 crypts per genotype. *P*= 0.2432 (two tailed t-test).

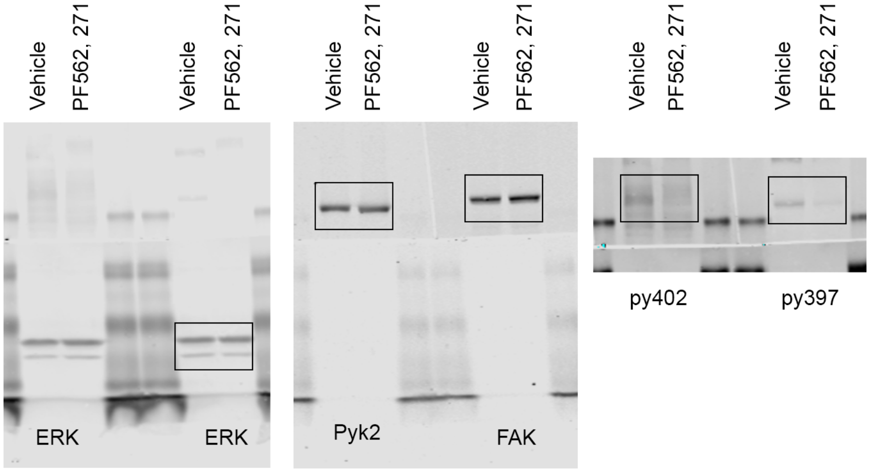

Supplementary Fig. S4. Original immunoblots corresponding to Figure 3. Intestinal organoids were treated with either vehicle or inhibitor (PF562,271) as described in Methods. Organoids were then harvested, lysed and immunoblotted for total ERK (left), total FAK or Pyk2 (center), phospho-Pyk2 (pY402) or phospho-FAK (pY397). Areas that were cropped and used in Fig. 3 are boxed.
